# *JAK2*V617F variant allele frequency*,* non-driver mutations, single-nucleotide variants and polycythemia vera outcome

**DOI:** 10.1007/s00432-022-04327-0

**Published:** 2022-10-15

**Authors:** Zuzanna Kanduła, Michał Janowski, Barbara Więckowska, Edyta Paczkowska, Krzysztof Lewandowski

**Affiliations:** 1 Department of Hematology and Bone Marrow Transplantation, Poznań University of Medical Sciences, Poznań, Poland; 2 Department of General Pathology, Pomeranian Medical University, Szczecin, Poland; 3 Department of Computer Science and Statistics, Poznań University of Medical Sciences, Poznań, Poland

**Keywords:** Polycythemia vera, *JAK2*V617F VAF, Myelofibrosis free survival, Thrombosis, Blastic transformation risk, Death risk

## Abstract

**Introduction:**

Despite comparatively favourable prognosis in polycythemia vera (PV) patients (pts), the overall survival is shorter compared to the age-matched general population. The aim of the study was to evaluate the impact of chosen laboratory and genetic factors on the individual disease outcome, i.e. risk of thrombosis, myelofibrosis/blastic transformation and death.

**Materials and methods:**

The study group consisted of 151 pts and 57 healthy donors (HD).

**Results:**

*JAK2*V617F mutation was found in 96.7% (146/151) of the studied pts. *JAK2* exon 12 mutations were identified in 2 individuals. The coexistence of *JAK2*V617F and *JAK2* exon 12 mutation was confirmed in 2 other pts. In one case, neither *JAK2*V617F nor *JAK2* exon 12 mutation was found. The presence of ten different non-driver mutations (*ASXL1, SRSF2, U2AF1, IDH2*) in eight of the analyzed pts (5.3%) was confirmed. The overall frequency of thrombotic events (TE) in the studied PV group was 23.8% (36/151). In patients with TE, median platelet count was lower than in pts without TE. Thrombotic risk did not depend on *JAK2* rs12343867, *TERT* rs2736100, *OBFC1* rs9420907 SNV, however, we found a novel strong tendency towards statistical significance between the CC genotype *miR-146a* rs2431697 and thrombosis. The disease progression to fibrotic phase was confirmed in 9% of the pts. Fibrotic transformation in PV pts was affected mainly by *JAK2*V617F variant allele frequency (VAF) and the presence of coexisting non-driver variants. The high *JAK2*V617F VAF and elevated white blood cell (WBC) count at the time of diagnosis were associated with an increased risk of death.

**Conclusion:**

Therefore, in our opinion, complex, laboratory and genetic PV pts evaluation at the time of diagnosis should be incorporated into a new prognostic scoring system to more precisely define the PV prognosis and to optimize the therapeutic decision-making process.

**Supplementary Information:**

The online version contains supplementary material available at 10.1007/s00432-022-04327-0.

## Introduction

Polycythemia vera (PV) together with essential thrombocythemia (ET) and primary myelofibrosis (PMF) is classified as a classical Philadelphia negative myeloproliferative neoplasm (MPN Ph-) (Arber et al. 2016). Among various registries, the annual incidence of PV is estimated at 0.4–2.8 per 100.000 and the prevalence at 1/3.300, with a male/female ratio of 1.2:1. PV occurs at all ages but is most common in subjects aged 50–70 years (Moulard et al. 2014; Shallis et al. 2020a). The disease is mainly characterized by an increase of red blood cells mass and content; an increase of haemoglobin and haematocrit value, and thrombotic tendency, including microvascular complications. Thrombotic episodes at the time of diagnosis are relatively frequent and are observed in 12 to 39% of PV patients (pts) (Tefferi and Elliott 2007; Falanga and Marchetti 2012). More frequently, the microvascular complications occurs, including numbness (66.2%), concentration difficulties (61.2%), sad mood (65%) and sexuality difficulties (56.8%), headache (52.2%), and dizziness (52.1%) (Cuthbert and Stein 2019).

Initial reports documented that 95% of PV pts carry the V617F mutation (exon 14) of the *JAK2* gene, resulting in a diminished JH2 inhibitory effect on the adjacent JH1 kinase domain, thus keeping JAK2 in a constitutively active conformation (Chen and Mullally 2014). Most of the *JAK2*V617F negative cases involves *JAK2* exon12 mutations (different in nature-synonymous substitutions, deletion variants, duplications) (Scott 2011). In small number of PV pts, the driving mutation leading to the clinical symptoms occurrence has been not identified yet. The *JAK2*V617F VAF was shown to have laboratory and clinical implications (Borowczyk et al. 2015). Its quantification, however, is not obligatory for the diagnosis nor the thrombotic risk assessment of patients with PV according to the WHO criteria. It has been shown that *JAK2*V617F homozygosity positively correlates with erythropoiesis and granulopoiesis, splenomegaly, pruritus, requiring cytoreductive therapy and negatively correlates with the platelet (PLT) count (Vannucchi et al. 2007a). In comparison to the *JAK2*V617F positive pts, the individuals carrying the *JAK2* exon 12 mutation are characterised at the time of diagnosis by younger age, higher Hb level, lower WBC and PLT count. Despite the differences in the laboratory patients’ characteristics, the rates of survival and constitutive symptoms are similar to patients carrying the *JAK2*V617F mutation (Passamonti et al. 2011; Tondeur et al. 2021). However, the molecular landscape of non-driver mutations in not as homogenous as driver mutations. The question of the impact of *JAK2*V617F VAF, single-nucleotide variants (SNVs) and the coexisting non-driver variants on the individual PV disease course (risk of: thrombosis, fibrotic/blastic progression, death) is still not fully answered.

So far *ASXL1*, *SRSF2*, *IDH2* have been identified as unfavourable risk factors affecting overall (OS), leukemia-free (LFS), or myelofibrosis-free survival (MFFS) in PV (Tefferi et al. 2016). In 2020, the *SRSF2* mutation positivity has been incorporated in the prognostic scoring systems-MIPSS-PV, as an independent genetic risk factor in PV, together with age > 67 years, leukocyte count ≥ 15G/L, and previous thrombosis (Tefferi et al. 2020). A lot of effort has been made to better understand the PV biology and to identify risk factors associated with the unfavourable PV outcome at the time of diagnosis. The risk factors associated with thrombosis in PV have been not fully recognized. The data suggest a relevant role of regulatory miRNAs involved in the platelet activation process and immune system control (Krammer et al. 2022). Among others, the *miR-146a,* a brake in NF-κB signaling, has been shown as a key mediator of inflammation-induced carcinogenesis and in thromboinflammation (Boldin et al. 2011; Arroyo et al. 2021).

Herein, we present the results of our study evaluating the impact of the factors postulated to be associated with PV occurrence, manifestation and outcome, including the SNVs’ genotypes (*JAK2* rs12343867, *TERT* rs2736100, *OBFC1* rs9420907, *miR-146a* rs2431697) and non-driver genetic variants (*SRSF2, ASXL1, U2AF1, IDH1, IDH2*). Special attention was paid to their impact on the risk of thrombosis, fibrotic progression, blastic transformation, and death of the evaluated patients.

### Study group characteristics

The study group consisted of 151 pts recruited from the 2 Polish university centres - the Department of Hematology and Bone Marrow Transplantation of Poznan University of Medical Sciences in Poznan and the Department of Hematology of Pomeranian Medical University in Szczecin, and 57 healthy donors (control group). The diagnosis of PV, post-PV-MF (post-PV-myelofibrosis) and blastic phase was established according to the WHO criteria published in 2016 (Arber et al. 2016). The grade of the bone marrow fibrosis was assessed according to the European Consensus on grading bone marrow fibrosis and the assessment of cellularity (Thiele et al. 2005). A characteristic of the patients studied is presented in (Table 1). The median follow-up was 60 months [0–240]. 27 patients died between the time of diagnosis and the end of the study. This study was conducted in accordance with the Declaration of Helsinki. The study protocol was approved by the Ethics Committee of Poznan University of Medical Sciences (number: 1056/16, 181/18 and 846/21).

**Table 1:** Main characteristic of PV patients included in the study

	*n* = 151	%
Male/female	64/87	(42.4/57.6)
Median age at time of the diagnosis, y [range]	60 [20–88]	–
*JAK2*V617F positive	146	96.7
*JAK2 exon 12* positive	2	1.3
*JAK2*V617F *and JAK2 exon 12* positive	2	1.3
*JAK2*V617F *and JAK2 exon 12* negative	1	0.7
Patients carrying non-driver variants	8	5.3
–Number of variants	10	–

## Materials and methods

DNA was extracted from whole-blood leukocytes at the time of diagnosis or first evaluation at our Department using QIAmp DNA Mini Kit (Qiagen). The assessment for the presence of the *JAK2*V617F mutation was conducted by quantitative allele-specific RQ-PCR according to Larsen et al. (2007) standardized by cooperation with MPN&MPNr EuroNet (Jovanovic et al. 2013). High-resolution melt analysis (HRMA) was used to detect the following variants: *JAK2* (exon 12 and 14), *SRSF2* (exon1)*, U2AF1* (exon 2 and 6)*, IDH1* (exon 4), *IDH2* (exon 4)*, TERT* rs2736100*, OBFC1* rs9420907*,* miR-146a rs2431697 and *JAK2* rs12343867 (SNV in complete linkage disequilibrium with the 46/1 haplotype). For the variant’s type identification screened by HRMA, the Sanger sequencing was applied, using the BigDye Terminator v3.1 Cycle Sequencing kit (Applied Biosystems, Thermo Fisher Scientific). Similarly, Sanger sequencing was applied for the exon 13 (range Ile574 to Glu727) of *ASXL1* gene analysis (a region covering at least 83% of all known *ASXL1* mutations) (Gelsi-Boyer et al. 2009; Pratcorona et al. 2012). PCR primers sequence and the details of the method are listed in Supplementary Table 1.

### Statistical analysis

Nominal data were described using counts and percentages for each category. A comparison of such data between the study and control group was performed using the chi-square test or its correction (Fisher's exact test) when the numbers in individual categories were too low. In the case of variables with several categories, when the effect obtained was statistically significant, multiple comparisons were also applied using the chi-square test and Fisher’s exact test, but adding the Benjamini–Hochberg correction. In addition, to describe the magnitude of the obtained effect, an odds ratio was determined together with a 95% confidence interval, giving the chance of occurrence of the event in the exposed group in relation to the reference category. A Cochran-Armitage chi-square test for trend was calculated to look for a trend in the obtained proportions. *JAK2*V617F VAF was presented in four equally sized categories defined by the boundaries: 0%; 25%; 50%; 75%; 100%. Continuous data were described using mean ± standard deviation and median with quartiles. Comparisons of continuous variables according to normality of distribution and equality of variance (tested by Shapiro–Wilk test and Fisher-Snedecor test, respectively) were made using unpaired *t* test or exact version of Mann–Whitney test dedicated to small sample sizes. The strength and direction of the association between *JAK2*V617F % and continuous variables were assessed using Spearman’s monotonic correlation coefficient with 95% confidence interval and the test of its statistical significance. The survival analysis was performed using Kaplan–Meier curves which were compared with the log-rank test. The predictive ability for individual parameters was examined using receiver operating characteristic (ROC) curves. The size of the area under the curve (AUC) was determined along with the 95% confidence interval, the cut-off point (via Youden’s index) for the studied parameters was identified, and the sensitivity and specificity values for it were given. All analyses were performed in PQStat v1.8.4 software. A significance level of 0.05 was assumed.

## Results

### Molecular characteristic of studied patients

#### *JAK2* mutation and non-driver variants frequency

The presence of *JAK2*V617F mutation was confirmed in 146 out of the 151 pts (96.7%). The *JAK2* exon12 mutations were identified in 2 (1.3%) individuals. The coexistence of *JAK2*V617F and *JAK2* exon12 mutation was confirmed in 2 (1.3%) other pts. In one case (0.7%) neither *JAK2*V617F nor *JAK2* exon12 mutation was found (Table 1). Additionally, the presence of ten different non-driver mutations (*ASXL1, SRSF2, U2AF1, IDH2*) in eight of the analyzed pts (8/151; 5.3%) was confirmed (Table 2). No *IDH1* variant was identified. Interestingly, in case of *JAK2* exon12 positive pts or pts with coexisting *JAK2*V617F and *JAK2* exon 12 mutations, non-driving gene aberrations were not found.

**Table 2:** Molecular characteristics of PV pts with the presence of coexisting non-driver variants

Patient’s ID	*JAK2*V617F VAF (%)	*ASXL1* (exon 13)	*SRSF2* (exon 1)	*U2AF1* (exon 2 and 6)	*IDH1* (exon 4)	*IDH2* (exon 4)
PV_27	30	–	–	c.470G > A p.Gln157Arg	–	–
PV_30	59	c.1720-2A > G	c.284C > A p.Pro95His	–	–	–
PV_36	71	c.1934dup p.Gly646Trpfs*12	–	–	–	–
PV_64	80	c.2077C > T p.Arg693Ter	–	–	–	–
PV_109	89	c.1934dup p.Gly646Trpfs*12	–	–	–	c.419G > A p.Arg140Gln
PV_146	47	c.1900_1922del p.Glu635ArgfsTer15	–	–	–	–
PV_161	12	c.1984_1985del p.Gly662GlnfsTer5	–	–	–	–
PV_165	62	c.2135_2139del p.Ala712ValfsTer4	–	–	–	–

#### SNVs’ frequency

The distribution of the different SNVs’ genotypes (*JAK2* rs12343867 T>C, *TERT* rs2736100 A >C, *OBFC1* 9420907 A> C and *miR-146a* rs2431697 C >T) in PV patients and healthy donor groups is presented in Table 3. Statistically significant differences in SNVs’ genotypes frequency between PV and control group were found in the case of *JAK2* rs12343867 (*p* = 0.0001) and *TERT* rs2736100 (*p* < 0.0001). The differences were evident for each of the genotype subgroups, i.e. for *JAK2* rs12343867 TT/TC/CC and *TERT* rs2736100 AA/AC/CC, which was confirmed by the Benjamini–Hochberg corrected multiple comparison. The risk of PV is the greatest and lowest in the case of *JAK2* rs12343867 in CC and TT individuals, respectively, and in the case of *TERT* rs2736100 in CC and AA individuals, respectively.

**Table 3:** Frequency of SNVs’ genotypes in PV patients and healthy donors group

SNV	PV		Control		*p*-value*	*p*-value^#^	OR^$^ [95% CI]	*p*-value^*B-H*^			
	*n*	%	*n*	%							
*JAK2* rs12343867					*0.0001* ^*B-H*^*CC* > *CT* > *TT*	< *0.0001*		*JAK2* rs12343867	CC	CT	TT
CC	48	(32)	5	(8)			8.80 [2.97; 26.11]	CC		0.0041	0.0001
CT	79	(52)	35	(56)			2.07 [1.03; 4.18]	CT	0.0041		0.0406
TT	24	(16)	22	(35)			Reference	TT	0.0001	0.0406	
*TERT* rs2736100					< *0.0001* ^*B-H*^*CC* > *AC* > *AA*	< *0.0001*		*TERT* rs2736100	AA	AC	CC
AA	14	(9)	28	(49)			Reference	AA		< 0.0001	< 0.0001
AC	81	(54)	23	(40)			7.04 [3.19; 15.54]	AC	< 0.0001		0.0412
CC	56	(37)	6	(11)			18.67 [6.48; 53.80]	CC	< 0.0001	0.0412	
*OBFC1* rs9420907					0.2445	0.1743					
CC	12	(8)	1	(2)			5.00 [0.63; 39.75]				
CA	43	(28)	16	(28)			1.12 [0.57; 2.22]				
AA	96	(64)	40	(70)			Reference				
*miR-146a* rs2431697					0.6441	0.5665					
TT	46	(31)	21	(37)			0.83 [0.35; 1.97]				
TC	76	(50)	25	(44)			1.15 [0.50, 2.64]				
CC	29	(19)	11	(19)			Reference				

^*^chi-square test

^#^Cochran–Armitage test for trend

^$^Odds Ratio with 95% confidence interval

^*B*^^*−*^^*H*^Benjamini-Hochberg corrected multiple comparison

### PV manifestation and outcome

#### *JAK2*V617F VAF and complete blood count results

*JAK2*V617F VAF correlated positively with total WBC count, neutrophil (NEU) count, red cell distribution width (RDW), and negatively with PLT count, mean cell haemoglobin (MCH), mean cell haemoglobin concentration (MCHC) (Supplementary Table 2)*. JAK2*V617F VAF was higher in PV * JAK2*V617F positive patients with coexisting variants than in the *JAK2*V617F mutation positive only (61 vs 26%. *p* = 0.0121). No significant differences were found in terms of complete blood count parameters between the groups with and without coexisting variants (Supplementary Table 3).

#### PV and thrombosis

The overall frequency of thrombotic events in the studied PV group was 23.8% (36/151). In 12 (33.3%) patients, thrombotic episodes occurred before the disease diagnosis (6–venous, 6–arterial) and in 24 (66.7%) individuals after the PV treatment initiation (17–venous, 7–arterial). There was no death due to thrombotic complications. Thrombotic events risk did not depend on the genotypes of *JAK2* rs12343867, *TERT* rs2736100, *OBFC1* rs9420907 (Table 4), *JAK2*V617F VAF nor the presence of additional variants (Table 5). However, it is worth mentioning that in the case of *miR-146a* rs2431697, the obtained *p*-value was 0.0511 (Cochran–Armitage test for trend) and the obtained odds ratios were more than twice lower in the CT and TT groups, as compared to CC. In patients with and without thrombotic event, the median platelet count was 436 [139–1171] and 539 [43–1411] G/L, respectively, and the frequency of TT/TC/CC genotype was 19%/44%/36% and 29%/52%/19%, respectively. According to the TT/TC/CC genotype, the platelet count was 446 [160–932], 477 [43–1411] and 574 [212–1171] G/L, respectively, in the whole study group.

**Table 4:** The impact of the studied SNVs’ genotypes on the risk of thrombosis in the studied PV pts group

Thrombosis	Yes		No		*p-*value*	*p-*value^#^	OR^$^ [95% CI]
SNV	*n*	%	*n*	%			
*JAK2* rs12343867					0.4532	0.4722	
CC	14	(39)	33	(29)			1.27 [0.42; 3.88]
CT	16	(44)	64	(56)			0.75 [0.26; 2.20]
TT	6	(17)	18	(16)			Reference
*TERT* rs2736100					0.9546	0.7617	
AA	3	(8)	11	(10)			Reference
AC	19	(53)	62	(54)			1.12 [0.28; 4.45]
CC	14	(39)	42	(37)			1.22 [0.3; 5.02]
*OBFC1* rs9420907					0.6714	0.7583	
CC	4	(11)	8	(7)			1.59 [0.44; 5.76]
CA	9	(25)	34	(30)			0.84 [0.35; 2.01]
AA	23	(64)	73	(63)			Reference
*miR-146a* rs2431697					0.1112	0.0511	
TT	7	(19)	33	(29)			0.36 [0.12; 1.04]
TC	16	(44)	60	(52)			0.45 [0.19; 1.09]
CC	13	(36)	22	(19)			Reference

*Fisher exact test

^#^Cochran–Armitage test for trend

^$^Odds Ratio with 95% confidence interval

**Table 5:** The impact of the additional non-driver variants on the thrombosis, fibrotic-/blastic transformation risk in the studied PV patients

Additional variant	Yes		No		*p*-value*	OR^$^ [95%CI]
	*n*	%	*n*	%		
Thrombosis					1	
Yes	2	(25)	34	(24)		0.94 [0.18; 4.85]
No	6	(75)	109	(76)		Reference
Fibrotic transformation (*n* = 73)					*0.0029*	
Yes	4	(20)	9	(13)		26.22 [2.63; 261.77]
No	1	(80)	59	(87)		Reference
Blastic transformation					0.1516	
Yes	1	(13)	2	(1)		10.07 [0.81; 124.88]
No	7	(88)	141	(99)		Reference

*Fisher exact test

^$^Odds Ratio with 95% confidence interval

#### PV progression to the fibrotic phase

**Fig. 1:**
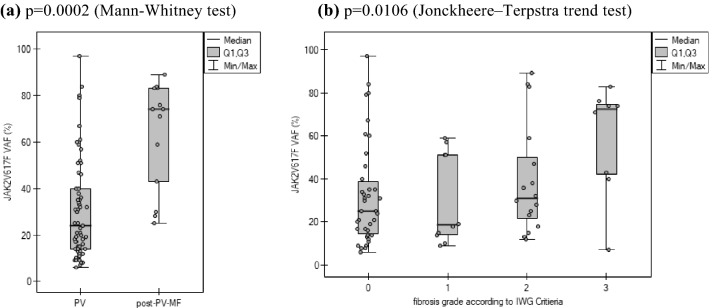
The *JAK2*V617F variant allele frequency at diagnosis time and the bone marrow fibrosis grade (only patients with trephine biopsy performed included) according to **a** disease stage **b** IWG Criteria

The bone marrow trephine biopsy was performed in 73 of PV pts, with the clinical suspicion of disease progression to the fibrotic phase (the presence of at least one symptom of the disease progression, like anemia or sustained loss of requirement of either phlebotomy in the absence of cytoreductive therapy or cytoreductive treatment for erythrocytosis; a leukoerythroblastic peripheral blood picture; increasing splenomegaly defined as either an increase in palpable splenomegaly of ≥ 5 cm or the appearance of a newly palpable splenomegaly; development of ≥ 1 of three constitutional symptoms: > 10% weight loss in 6 months, night sweats, unexplained fever > 37.5 °C). The disease progression to the fibrotic phase was confirmed in 9% (13/151) of the studied pts with clinical suspicion of disease evolution. The median follow-up was 60 [0–420] and 228 [24–348] months in PV and in post-PV-MF, respectively. Median MFFS time was established to 240 months. It should be stressed, however, that in 11 other pts qualified for trephine biopsy, the fibrosis grade ≥ 2 was found, but the post-PV-MF was not diagnosed due to the non-fulfillment of the other obligatory IWG Criteria (Barosi et al. 2008). Median *JAK2*V617F VAF at the time of PV diagnosis was 74 and 24% in patients who progressed and who did not progress to post-PV-MF, respectively (*p* = 0.0002) (Fig. 1a). The fibrosis grade depending on the baseline *JAK2*V617F VAF and Kaplan–Meier curves for MFFS time are presented in Fig. 1b and Supplementary Fig. 1, respectively. The *JAK2* rs12343867 CC genotype (Table 6) and the presence of coexisting non-driver variant (Table 5) were identified as strongly associated with an increased risk of post-PV-MF development.

**Table 6:** The SNVs’ impact on the disease progression risk to fibrotic phase in the studied PV pts

Post-PV-MF	Yes		No		*p*-value*	*p*-value^#^	OR^$^ [95%CI]	*p*-value^*B*^^*−*^^*H*^			
SNV	*n*	%	*n*	%							
*JAK2* rs12343867					*0.0001* ^*B-H*^* CC* > *CT*	*0.0005*		*JAK2* rs12343867	CC	CT	TT
CC	11	(85)	36	(26)			Reference	CC		0.0002*	0.0732*
CT	1	(8)	79	(57)			0.04 [0.01; 0.33]	CT	0.0002*		0.4100*
TT	1	(8)	23	(17)			0.14 [0.02; 1.18]	TT	0.0732*	0.4100*	
*TERT* rs2736100					0.4217	0.2657					
AA	1	(8)	13	(9)			Reference				
AC	5	(38)	76	(55)			0.86 [0.09; 7.92]				
CC	7	(54)	49	(36)			1.86 [0.21; 16.47]				
*OBFC1* rs9420907					0.5641	0.5748					
CC	2	(15)	10	(7)			2.20 [0.41; 11.83]				
CA	3	(23)	40	(29)			0.83 [0.21; 3.27]				
AA	8	(62)	88	(64)			Reference				
*miR-146a* rs2431697					0.5747	0.6644					
TT	6	(46)	20	(33)			1.20 [0.25; 5.71]				
TC	4	(31)	28	(47)			0.57 [0.11; 2.95]				
CC	3	(23)	12	(20)			Reference				

*Fisher exact test

^#^Cochran–Armitage test for trend

^$^Odds Ratio with 95% confidence interval

^*B*^^*−*^^*H*^Benjamini-Hochberg corrected multiple comparison

Genotype analysis did not confirm the impact of *miR-146a* rs2431697, a brake in NF-κB signaling and a key mediator of inflammation-induced carcinogenesis, on the risk of PV progression to the fibrotic phase.

#### PV progression to the blastic phase

Three out of 151 of PV patients (2%) progressed to the blastic phase during the observation follow-up. The risk of blastic transformation was ten times higher in patients with coexisting non-driver genetic variants (Table 5). A molecular characteristic of the PV patients transformed to the blastic phase is shown in Table 7.

**Table 7:** Molecular characteristic of pts progressed to blastic phase

Patient’s ID	*JAK2*V617F VAF (%)	*JAK2 *exon12	*ASXL1*	*SRSF2*	*U2AF1*	*IDH1*	*IDH2*	LFS (months)
PV_107	62	–	–	–	–	–	–	264
PV_109	89	–	c.1934dup p.Gly646Trpfs*12	–	–	–	c.419G > A p.Arg140Gln	312
PV_153	0.12	c.1627_1632del p.Glu543_Asp544del	–	–	–	–	–	24

#### Overall survival of the PV patients

The risk of death in the studied patient group was closely associated with *JAK2*V617F VAF at the time of diagnosis. A detailed analysis confirmed a simple association: the higher VAF, the lower chance of survival (*p* = 0.0028) (Table 8). The association between the different SNVs’ genotypes and OS time are presented in Table 9 and in Supplementary Fig. 2, respectively. *JAK2* rs12343867 CC genotype is associated with an increased risk of death than CT.

**Table 8:** The *JAK2*V617F VAF and the risk of death of PV patients

*JAK2* V617F (%) [range]						*JAK2* V617F (%) [range]				
	Death		*p*-value*	*p*-value^#^	OR [95% CI]	^*B-H*^*p*-value				
	Yes	No								
			0.0028 ^*B-H*^ (50,75] < reference (75,100] < reference	0.0002			(75, 100]	(50, 75]	(25, 50]	(0, 25]
(75, 100]	5	6			8.75 [2.05; 37.40]	(75, 100]		0.7077*	0.0749*	0.0212*
(50, 75]	8	16			5.25 [1.59; 17.30]	(50, 75]	0.7077*		0.1029	0.0212*
(25, 50]	6	36			1.75 [0.53; 5.83]	(25, 50]	0.0749*	0.1029		0.4380*
(0, 25]	6	63			reference	(0, 25]	0.0212*	0.0212*	0.4380*	

*Fisher exact test

^#^Cochran–Armitage test for trend

^$^Odds Ratio with 95% confidence interval

^*B*^^*−*^^*H*^Benjamini-Hochberg corrected multiple comparison

**Table 9:** SNVs’ genotypes and the death risk in PV patients

Death	Yes		No		*p*-value */**	*p*-value^#^	OR^$^ [95% CI]
SNV	*n*	%	*n*	%			
*JAK2* rs12343867					0.0528**	0.2169	
CC	13	(48)	34	(27)			Reference
CT	9	(33)	71	(57)			0.33 [0.13; 0.85]
TT	5	(19)	19	(15)			0.69 [0.21; 2.23]
*TERT* rs2736100					0.5716*	0.6106	
AA	3	(11)	11	(9)			Reference
AC	12	(44)	69	(56)			0.64 [0.15; 2.63]
CC	12	(44)	44	(35)			1.00 [0.24; 4.17]
*OBFC1* rs9420907					0.8267*	0.7338	
CC	2	(7)	10	(8)			1.00 [0.20; 5.00]
CA	9	(33)	34	(27)			1.32 [0.53; 3.29]
AA	16	(59)	80	(65)			Reference
*miR-146a* rs2431697					0.9380*	0.7694	
TT	9	(33)	37	(30)			1.17 [0.35; 3.91]
TC	13	(48)	63	(51)			0.99 [0.32; 3.08]
CC	5	(19)	24	(19)			Reference

*Chi-square test or

**Fisher exact test if appropriate

^#^Cochran–Armitage test for trend

^$^Odds Ratio with 95% confidence interval

#### Factors associated with unfavourable PV outcome by ROC analysis

**Fig. 2:**
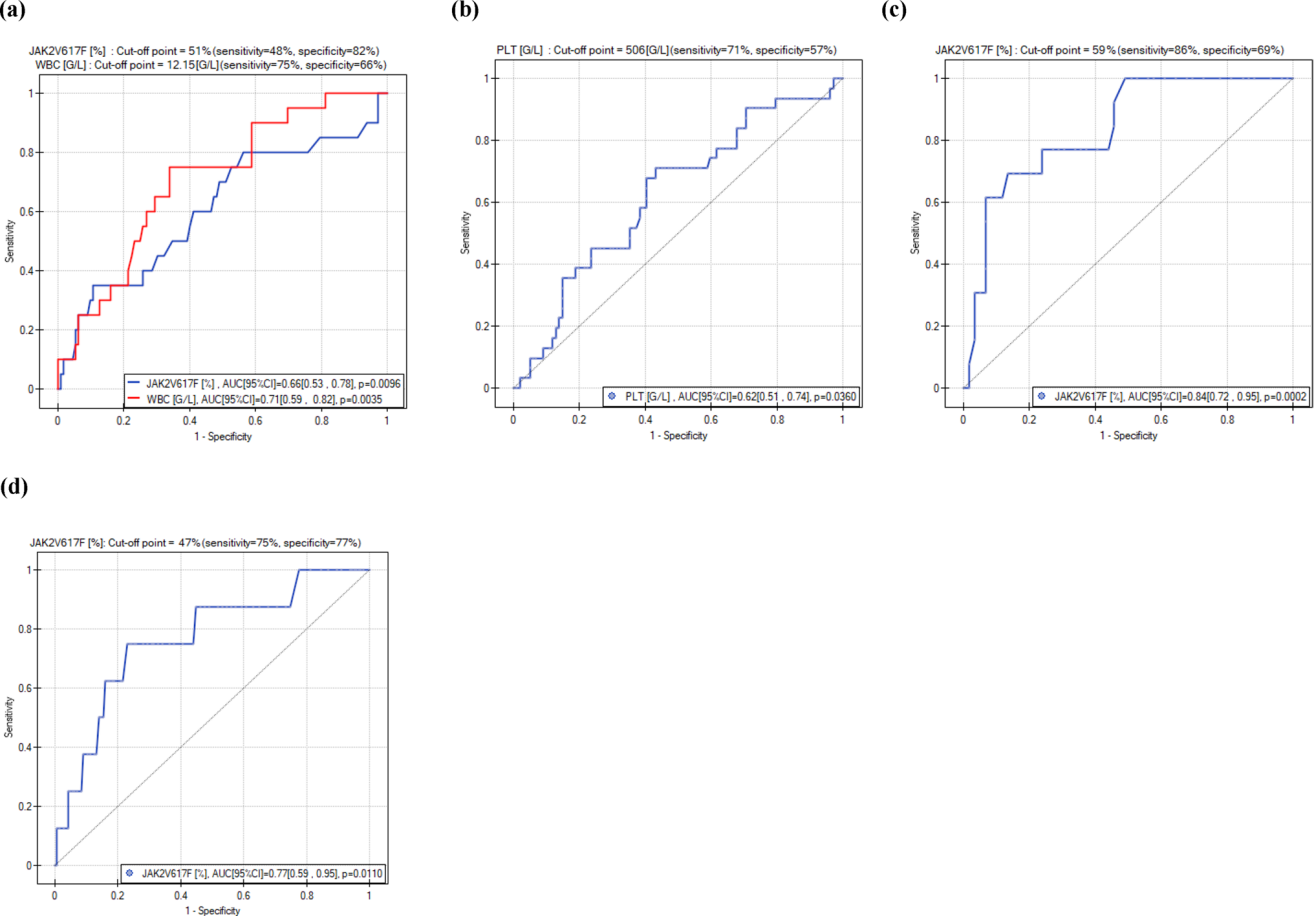
The ROC curves of the factors influencing the PV outcome **a** the *JAK2*V617F VAF and WBC as death predictors **b** the PLT count as thrombosis predictor **c** *JAK2*V617F VAF as predictor of PV evolution to fibrotic phase (post-PV-myelofibrosis) **d** *JAK2*V617F as predictor of coexisting variants

The ROC curve analysis including *JAK2*V617F VAF, WBC, NEU, LYM, MONO, EOS, BASO, LUC, RBC, HB, HT, MCV, MCH, MCHC, MCHC, RDW, PLT, and MPV revealed that only higher *JAK2*V617F VAF (AUC[95% CI] = 0.66[0.53;0.78], *p* = 0.0096, cut-off point = 51%) and higher WBC (AUC[95% CI] = 0.71[0.59; 0.82], *p* = 0.0035, cut-off point = 12.15 G/L) at the time of diagnosis could serve as predictor of preterm death of patients with PV. A similar analysis showed that a lower PLT count (AUC[95% CI] = 0.62[0.51;0.74], *p* = 0.0360, cut-off point = 506 G/L) could be used as thrombotic predictor. The strongest association in the ROC curve analysis was shown between the higher *JAK2*V617F VAF and post-PV-MF risk (AUC[95% CI] = 0.84[0.72;0.95], *p* = 0.0002, cut-off point = 59%) (Fig. 2). For *JAK2*V617F, the specificity is higher (the ability to exclude the preterm death), while for WBC, the sensitivity is higher (the ability to detect patients who will die). Moreover, the occurrence of coexisting variants is associated with higher *JAK2*V617F VAF (AUC[95% CI] = 0.77[0.59;0.95], *p* = 0.0110, cut-off point = 47%).

## Discussion

The prognosis in PV pts is comparatively favourable, but individual disease course is very heterogeneous. It is mainly due to the different risk of thrombosis and disease evolution to the fibrotic or blastic phase. The overall survival of PV patients is shorter compared to the age-matched general population (Srour et al. 2016; Abu-Zeinah et al. 2022), superior to that in PMF and inferior to that in ET patients, with estimated median OS of 11.9–14.0, 4.0–6.0 and 12.1–20.0 years, respectively (Tefferi et al. 2014; Shallis et al. 2020b; Smith et al. 2021). According to Kiladjian et al., the risk of progression to the fibrotic phase is 4.9 and 9.4% after 10 and 15 years, and according to Bonicelli et al., 6, 14 and 26% after 10, 15 and 20 years, respectively (Kiladjian et al. 2003; Bonicelli et al. 2013). The rates of leukemic transformation are 1.0–3.9% in PV, 1.5–2.6% in ET and 3.3–9.2% in PMF (Szuber et al. 2019; Shallis et al. 2020b; Smith et al. 2021). In the case of PV blastic transformation (secondary acute myeloid leukemia), the reported OS is about 4 months (Abdulkarim et al. 2009). According to the Weill Cornell Medicine (PV-WCM) Research Database Repository data, an excess late mortality was observed after 17 years, potentially due to an increased incidence of post-PV-MF progression, affecting almost 50% of PV pts after 25 years (Abu-Zeinah et al. 2022). On the basis of the Surveillance, Epidemiology, and End Results (SEER) Registry [*n* = 3840 pts] data analysis, the most frequent causes of death in PV include cardiovascular disease (26.1%), solid tumors (12.7%), other malignancies (12.6%), cerebrovascular disease (6%) myeloid malignancies (5%) infection (4.6%) and non-myeloid hematologic malignancies (1.3%) (Smith et al. 2021).

The above-mentioned data support the hypothesis that early identification of the factors influencing the individual risk of thrombosis, fibrotic/blastic transformation and death in PV patients may be very helpful in the decision-making process of treatment individualization. It has been postulated that individual genetic profile may be responsible for the differences in individual disease outcome. It was shown that 46/1 haplotype (Jones et al. 2009; Olcaydu et al. 2009; Anelli et al. 2018) and *TERT* rs2736100 A > C genotype predisposed to MPN (Oddsson et al. 2014). Lastly, Giaccherini et al., using a combined “teloscore” from 11 telomere-length-associated SNPs, indicated that genetically determined longer telomeres were a risk marker for the MPN development and also reported a novel association of the *OBFC1* rs9420907 C allele and increased MPN risk (Giaccherini et al. 2020). Our result confirm that *JAK2* rs12343867 CC and *TERT* rs2736100 CC are strongly correlated with PV predisposition, as was previously established, but contrary to other reports, we did not find the association between *OBFC1* rs9420907 CC genotype nor C allele and PV predisposition (Jones et al. 2009; Olcaydu et al. 2009; Anelli et al. 2018).

In 2020, Ferrer-Marin et al. and Aref et al. demonstrated that TT genotype of *miR-146a* rs2431697 polymorphism, correlated with inflammatory genes expression (IL-1β; NF-κB1 and NLRP3), was an early predictor of post-PV/ET-MF progression (Ferrer-Marín et al. 2020; Aref et al. 2021). Our results do not support this observation. This discrepancy may originate from the combined analysis of post-PV-MF and post-PV-ET patients (320 PV, 333 PV, 134 post-ET/PV-MF) in the first of the above-mentioned studies (Ferrer-Marín et al. 2020) and overrepresentation of post-PV-MF cases (40 PV vs 21 post-PV-MF) in the second one (Aref et al. 2021). In the available literature also, another polymorphism was associated with an unfavorable MPN Ph- outcome. The negative impact of the *JAK2* rs12343867 TT genotype on OS (poorer outcome) was also confirmed by Tefferi et al. in patients with PMF (Tefferi et al. 2019). Our results revealed the *JAK2* rs12343867 CC genotype was a risk factor of post-PV-MF development and shorter OS in comparison to CT but not TT genotype in PV patients.

Cardiovascular complications and cerebrovascular disease are a prominent cause of death, especially in younger patients with PV (Smith et al. 2021). The factors influencing the thrombotic risk in PV pts include age over 60 years and previous thrombosis. However, arterial hypertension, leukocytosis, and hypoxia should be considered as additional risk factors in certain circumstances (Stavik et al. 2016; Narita et al. 2016; Tefferi et al. 2018; Pilli et al. 2018).

We have been the first to find that PLT count lower than 506 G/L at the time of diagnosis is as a predictor of thrombosis. Additionally, we report a novel strong trend towards statistical significance between *miR-146a* rs2431697 CC genotype and thrombotic risk, as well. The above-mentioned association between the *miR*-146a rs2431697 CC genotype, thrombosis and platelet count seems to be important, especially evident in the light of 1) the discovery that rs2431697 C allele binds NF-κB more effectively than rs2431697 T allele, increasing the expression of mi-R146a (Hou et al. 2021), 2) the confirmation that mi-R146a activates JAK2/STAT3 (Wang et al. 2019) and 3) the documentation of the role of both NF-κB and mi-R146a in platelet signaling process, as reviewed by Kojok (Kojok et al. 2019) and Czajka (Czajka et al. 2021). It cannot be excluded that elevated *miR*-146a expression increases the thrombotic risk in PV pts by the impairment of fibrinolysis. The above-mentioned hypothesis is supported by the observation of Alfano et al., showing that overexpression of miR-146a reduces urokinase-type plasminogen activator receptor (uPAR) expression in AML cell lines (Alfano et al. 2015). Limited data are available on the role of fibrinolysis in the pathogenesis of thrombotic complications in MPN Ph- pts. Most of them postulated hypofibrinolysis as a prothrombotic mechanism in MPN Ph-. Specifically, hypofibrinolysis is driven by elevated levels of plasminogen activator inhibitor-1 (PAI-1) and platelet factor 4 (PF4). These proteins are associated with excessive platelet reactivity, not with the platelet count, which is in line with our results confirming the relationship between lower platelet count and thrombosis risk (Pósán et al. 1998; Birdane et al. 2005; Malecki et al. 2016). It should be stressed that activated platelets and leukocytes in ET patients were found to be the main source of tissue factor (TF) and that reduced activity of the TF pathway inhibitor additionally increases the prothrombotic risk in *JAK2*V617F positive ET patients (Gadomska et al. 2016).

Several studies have shown an association between leukocytosis and an increased risk of thrombosis (Landolfi et al. 2007; Gangat et al. 2007; De Stefano et al. 2010; Lim et al. 2015; Barbui et al. 2015; Cerquozzi et al. 2017; Carobbio et al. 2019). Data from the REVEAL study (prospective Observational Study of Patients with Polycythemia Vera in US Clinical Practices) signal that an elevated WBC count of more than 11 G/L increases the risk of thrombotic events and suggest the need to incorporate blood count values into the risk stratification (Gerds et al. 2021). Similar to Zhao et al., we did not find an association between the WBC count nor Hb level (Zhao et al. 2016). According to Ronner et al., the risk of thrombosis is not significantly associated with any hematologic laboratory value trajectory (Ronner et al. 2020). The results of our study suggest that rs2431697 CC genotype of the miR-146a, a brake in NF-κB signaling and a key mediator of inflammation-induced carcinogenesis and thromboinflammation, may be associated with thrombotic risk. The latter requires confirmation in larger groups of PV pts.

On the basis of the results of our study, we concluded that the WBC (≥ 12.15 G/L) at the time of diagnosis could serve as a predictor of preterm death in PV patients. This is in good compliance with the results obtained by Ronner et al. who found that persistently elevated leukocyte trajectories were significantly associated with an increased risk of unfavorable outcome of the disease due to the evolution to myelofibrosis, myelodysplastic syndrome, and acute myeloid leukemia (Ronner et al. 2020). For this reason, leukocytosis (≥ 15 G/L) has been determined to be an independent survival factor according to MIPSS-PV (Tefferi et al. 2020).

Another issue which is still debated is the impact of *JAK2*V617F VAF at the time of diagnosis on the individual disease outcome. *JAK2*V617F VAF at time of diagnosis in our patients was 31 and 20% in the case of venous and arterial thrombosis, respectively. Similar to Lee et al. (Lee et al. 2021), we did not confirm results which identified *JAK2*V617F VAF > 50% as an independent strong predictor of venous but not arterial thrombosis (Cerquozzi et al. 2017; Guglielmelli et al. 2021). Our results also documented that high *JAK2*V617F VAF at the time of diagnosis was associated with the risk of PV progression to post-PV-MF, which had been established previously by others (Vannucchi et al. 2007b; Passamonti et al. 2010; Alvarez-Larrán et al. 2014; Senín et al. 2018). The presence of such an association was neglected by Lee et al. (2021). Our results confirmed also that higher *JAK2*V617F VAF at the time of diagnosis was associated with impaired OS, as was also reported by Lee et al. (2021).

Complex molecular fingerprint seems to be an indispensable element of PV prognosis. It was documented that the presence of non-driver mutations in PV patients significantly changed the PV outcome. Until now, a large spectrum of coexisting somatic gene mutations affecting epigenetic regulation, messenger RNA splicing, signalling, transcriptional regulation and DNA repair in PV pts have been identified. In a study by Tefferi et al., the most frequently identified mutations in *JAK2*V617F positive pts were *TET2*, *ASXL1* and *SH2B3,* with the frequency of 22%, 12%, 9%, respectively. In a study by Song et al., the mutations of A*SXL1*, *KMT2A* and *TP53* coexisted with the driver mutation with the frequency of 6.25, 13.64 and 6.25%, respectively. Rarely, the presence of other gene mutations including splicing machinery genes such as *SRSF2*, *U2AF1*, *SF3B1* or *ZRSR2* has been confirmed (Tefferi et al. 2016; Song et al. 2017).

We detected 10 non-driver gene variants in 8 of 151 pts. Half of them experienced disease progression during the follow-up. Two pts - the first carrying the *U2AF1* and the second carrying the *ASXL1* variant, progressed to post-PV-MF after 184 and 200 months, respectively. The patient carrying the *IDH2* variant progressed to the fibrotic and blastic phase after 288 and 312 months of follow-up, respectively. The most rapid fibrotic progression we observed in a patient carrying the *ASXL1* splice variant and *SRSF2* variant (Kanduła et al. 2022). Four of our pts, carrying the *ASXL1* mutation, who have not progressed so far, were observed for 36, 48 and 180 months, respectively. When it comes to the analysis of *ASXL1* solely, we detected variants of this gene in 3% of pts who did not progress to post-PV-MF and in 23% who had, which it is in good agreement with results of Guo et al. (4 vs 26%) who demonstrated that PV patients with coexisting mutations of *ASXL1* had a poor MFFS (Guo et al. 2019). A study by Senín et al. indicates that the coexistence of additional genetic variants, especially in *SF3B1* and *IDH1/2*, is a strong predictor of MF transformation (Senín et al. 2018). According to the results of Lee et al., the *SF3B*1, *IDH1/2* and *ASXL1* variant occurrence is associated with fibrotic disease progression, and *RUNX1*, *TP53* and *IDH1/2* are associated with high leukemic transformation risk. *ASXL1*, *SRSF2*, *IDH2* were classified by Tefferi et al. as adverse variants/mutations, in terms of overall- (median survival 7.7 versus 16.9 years), leukemia-free- or myelofibrosis-free survival, with a combined prevalence of 15% (Tefferi et al. 2016). Bartels revealed that patients with *SRSF2, U2AF1,* and *IDH1/2* mutations at the time of diagnosis showed rapid transformation to the blastic phase and proposed to consider these mutations as negative predictors of rapid blastic progression in newly diagnosed MPN Ph- at the chronic stage (Bartels et al. 2021).

In conclusion, our study confirms the necessity of a complex clinical, laboratory and genetic PV patients work-up. It especially concerns the *JAK2*V617F VAF and *ASXL1*, *SRSF2*, *IDH2, U2AF1* mutation screening at the time of diagnosis to more accurately define the risk of progression to the fibrotic and/or blastic phase. The presence of the above-mentioned aberrations was confirmed by us in only 5.3% of PV patients. However, even in this case, the confirmation of their presence may improve the individual risk stratification and allow to optimize the therapeutic decision-making process in patients at risk of unfavourable disease outcome.

## Supplementary Information

Below is the link to the electronic supplementary material.

Supplementary file1 (DOCX 307 KB)

## Data Availability

The datasets generated during and/or analysed during the current study are available from the corresponding author on reasonable request.
