# Supplementary material for: JAK2V617F variant allele frequency, non-driver mutations, single-nucleotide variants and polycythemia vera outcome
Source: J Cancer Res Clin Oncol. 2022 Oct 15;149(8):4789–803. doi: 10.1007/s00432-022-04327-0 (PMC10349754; doi:10.1007/s00432-022-04327-0)

Supplementary Table 1
Primer sequences

| Gene/variant | Locus Genome Reference | Exon/intron | *Primer sequence 5’-3’* | | Product size [bp] | Annealing temperature [°C] |
| --- | --- | --- | --- | --- | --- | --- |
| *JAK2* V617F | LRG_612 | exon 14 | *F* | CTTTCTTTGAAGCAGCAAGTATGA | 101 | 60 |
|  |  |  | *P* | 6‐FAM‐TGAGCAAGCTTTCTCACAAGCATTTGGTTT‐TAMRA |  |  |
|  |  |  | *R_WT* | GTAGTTTTACTTACTCTCGTCTCCACAtAC |  |  |
|  |  |  | *R_MUT* | GTAGTTTTACTTACTCTCGTCTCCACAtAA |  |  |
| *JAK2* rs12343867 |  | intron 14 | *F* | ATGAAGACAAAGCATATAAATGATACA | 118 | 62 |
|  |  |  | *R* | AGTAGTTTCTGTGAACACCTAAA |  |  |
| *TERT* rs2736100 |  |  | F | CCCCACAAGCTAAGCATTAT | 152 | 62 |
|  |  |  | R | GAAGAACCACGCAAAGGAC |  |  |
| *OBFC1* rs9420907 |  |  | F | AACCAAACTTCTCAAATAGCACA | 200 | 62 |
|  |  |  | R | TTGAAGCAACAAGGCATTATACA |  |  |
| *miR-146a* rs2431697 |  |  | F | GGGGGTGAAAGAAGGAACTCA | 137 | 62 |
|  |  |  | R | CACGAGGCAAGCCAATGAAG |  |  |
| *SRSF2* | LRG_640 | exon 1 | F | CCCCTCAGCCCCGTTTACC | 212 | 62 |
|  |  |  | R | TTCGCCTTCGTTCGCTTTCA |  |  |
| *ASXL1* | LRG_630 | exon 13 | F | AGGTCAGATCACCCAGTCAGTT | 561 | 61 |
|  |  |  | R | TAGCCCATCTGTGAGTCCAACTGT |  |  |
| *IDH1* | LRG_610 | exon 4 | F | GGCTTGTGAGTGGATGGGTA | 90 | 60 |
|  |  |  | R | GCAAAATCACATTATTGCCAAC |  |  |
| *IDH2* | LRG_611 | exon 4 | F | GGGTTCAAATTCTGGTTGAA | 288 | 60 |
|  |  |  | R | TAGGCGAGGAGCTCCAGT |  |  |
| *U2AF1* | LRG_615 | exon 2 | F | GGGTGACGTCTCCCGAG | 189 | 60 |
|  |  |  | R | TCCCACCGCCTCAACCA |  |  |
|  |  | exon 6 | F | AATAATCAGCTCTCATTTTCCCT | 187 | 60 |
|  |  |  | R | ATGTAGAAATTAACTGTCTTTGAAAAGAAC |  |  |

Supplementary Table 2
CBC results in group without and with additional variants

|  |  | non-driver variant |  |  |  |
| --- | --- | --- | --- | --- | --- |
| Parameter |  | no (n=138) | yes (n=8) | p-value | statistical test |
| *JAK2* V617F% |  |  |  | *0.0121* | exact Mann-Whitney test |
|  | Mean ± SD | 32.12±22.88 | 56.25±25.71 |  |  |
|  | Median [ Q1; Q3 ] | 26[14; 43.75] | 60.5[42.75; 73.25] |  |  |
|  | Missing data | 0(0%) | 0(0%) |  |  |
| Range [JAK2 V617F%] |  |  |  |  |  |
|  | (75, 100] | 9(6.52%) | 2(25%) |  |  |
|  | (50, 75] | 21(15.22%) | 3(37.5%) |  |  |
|  | (25, 50] | 40(28.99%) | 2(25%) |  |  |
|  | (0, 25] | 68(49.28%) | 1(12.5%) |  |  |
| WBC G/L |  |  |  | 1 | exact Mann-Whitney test |
|  | Mean ± SD | 12.38±6.47 | 14.27±5.79 |  |  |
|  | Median [ Q1; Q3 ] | 10.7[8.42; 14.2] | 15.14[12.12; 17.29] |  |  |
|  | Missing data | 15(10.87%) | 4(50%) |  |  |
| NEU G/L |  |  |  | 1 | exact Mann-Whitney test |
|  | Mean ± SD | 7.84±3.34 | 9.35±3.86 |  |  |
|  | Median [ Q1; Q3 ] | 7.7[5.35; 9.36] | 9.63[7.55; 11.43] |  |  |
|  | Missing data | 41(29.71%) | 4(50%) |  |  |
| LYM G/L |  |  |  | 0.9129 | unpaired t-test |
|  | Mean ± SD | 1.84±0.68 | 1.81±0.76 |  |  |
|  | Median [ Q1; Q3 ] | 1.8[1.34; 2.18] | 1.57[1.45; 1.92] |  |  |
|  | Missing data | 49(35.51%) | 4(50%) |  |  |
| MONO G/L |  |  |  | 1 | exact Mann-Whitney test |
|  | Mean ± SD | 0.61±0.37 | 0.8±0.25 |  |  |
|  | Median [ Q1; Q3 ] | 0.54[0.42; 0.72] | 0.88[0.7; 0.94] |  |  |
|  | Missing data | 49(35.51%) | 5(62.5%) |  |  |
| EOS G/L |  |  |  | 1 | exact Mann-Whitney test |
|  | Mean ± SD | 0.39±0.81 | 0.26±0.24 |  |  |
|  | Median [ Q1; Q3 ] | 0.2[0.14; 0.42] | 0.28[0.15; 0.38] |  |  |
|  | Missing data | 49(35.51%) | 5(62.5%) |  |  |
| BASO G/L |  |  |  | 1 | exact Mann-Whitney test |
|  | Mean ± SD | 0.1±0.08 | 0.07±0.05 |  |  |
|  | Median [ Q1; Q3 ] | 0.08[0.04; 0.12] | 0.05[0.04; 0.09] |  |  |
|  | Missing data | 49(35.51%) | 5(62.5%) |  |  |
| LUC G/L |  |  |  | NA |  |
|  | Mean ± SD | 0.2±0.08 | NA±NA |  |  |
|  | Median [ Q1; Q3 ] | 0.18[0.15; 0.23] | NA[NA; NA] |  |  |
|  | Missing data | 89(64.49%) | 8(100%) |  |  |
| RBC T/L |  |  |  | 1 | exact Mann-Whitney test |
|  | Mean ± SD | 6.46±1.02 | 6.19±1.26 |  |  |
|  | Median [ Q1; Q3 ] | 6.35[5.82; 7.1] | 6.55[5.81; 6.92] |  |  |
|  | Missing data | 39(28.26%) | 4(50%) |  |  |
| HB MMOL/L |  |  |  | 1 | exact Mann-Whitney test |
|  | Mean ± SD | 10.73±1.23 | 10.12±0.43 |  |  |
|  | Median [ Q1; Q3 ] | 10.7[10.2; 11.43] | 10.3[10.05; 10.37] |  |  |
|  | Missing data | 14(10.14%) | 4(50%) |  |  |
| HT L/L |  |  |  | 1 | exact Mann-Whitney test |
|  | Mean ± SD | 0.53±0.06 | 0.5±0.05 |  |  |
|  | Median [ Q1; Q3 ] | 0.53[0.5; 0.56] | 0.51[0.49; 0.52] |  |  |
|  | Missing data | 22(15.94%) | 4(50%) |  |  |
| MCV fL |  |  |  | 0.9042 | unpaired t-test |
|  | Mean ± SD | 82.06±8.18 | 82.58±12.53 |  |  |
|  | Median [ Q1; Q3 ] | 81.8[76.55; 88] | 78.25[74.9; 85.93] |  |  |
|  | Missing data | 39(28.26%) | 4(50%) |  |  |
| MCH fmol |  |  |  | 1 | exact Mann-Whitney test |
|  | Mean ± SD | 1.64±0.25 | 1.69±0.33 |  |  |
|  | Median [ Q1; Q3 ] | 1.66[1.52; 1.8] | 1.58[1.48; 1.78] |  |  |
|  | Missing data | 39(28.26%) | 4(50%) |  |  |
| MCHC mmol/l |  |  |  | 0.8850 | unpaired t-test |
|  | Mean ± SD | 20.26±1.07 | 20.34±1.06 |  |  |
|  | Median [ Q1; Q3 ] | 20.16[19.67; 20.92] | 20.48[20.02; 20.79] |  |  |
|  | Missing data | 25(18.12%) | 4(50%) |  |  |
| RDW %' |  |  |  | 1 | exact Mann-Whitney test |
|  | Mean ± SD | 17.14±2.8 | 20.37±5.49 |  |  |
|  | Median [ Q1; Q3 ] | 16.8[14.95; 19.4] | 17.5[17.2; 22.1] |  |  |
|  | Missing data | 39(28.26%) | 5(62.5%) |  |  |
| PLT G/L |  |  |  | 1 | exact Mann-Whitney test |
|  | Mean ± SD | 566.87±263.85 | 399.75±208.74 |  |  |
|  | Median [ Q1; Q3 ] | 521[382.75; 699.75] | 325.5[299.5; 425.75] |  |  |
|  | Missing data | 14(10.14%) | 4(50%) |  |  |
| MPV fL |  |  |  | 0.1435 | unpaired t-test |
|  | Mean ± SD | 9.36±1.4 | 10.57±0.59 |  |  |
|  | Median [ Q1; Q3 ] | 9.5[8.3; 10.5] | 10.8[10.35; 10.9] |  |  |
|  | Missing data | 44(31.88%) | 5(62.5%) |  |  |

Supplementary Table 3
*JAK2* V617F and complete blood count

| Variables | | n missing data | n pairs | p-value | r (95%CI) |
| --- | --- | --- | --- | --- | --- |
| JAK2 V617F% | WBC G/L | 19 | 127 | *0.000001* | ***0,42 (0,26 ; 0,56)*** |
| JAK2 V617F% | NEU G/L | 45 | 101 | *0.000001* | ***0,46 (0,28 ; 0,6)*** |
| JAK2 V617F% | LYM G/L | 53 | 93 | 0.225876 | -0,13 (-0,33 ; 0,09) |
| JAK2 V617F% | MONO G/L | 54 | 92 | 0.788908 | 0,03 (-0,18 ; 0,24) |
| JAK2 V617F% | EOS G/L | 54 | 92 | 0.840902 | 0,02 (-0,19 ; 0,23) |
| JAK2 V617F% | BASO G/L | 54 | 92 | 0.406671 | 0,09 (-0,13 ; 0,29) |
| JAK2 V617F% | LUC G/L | 97 | 49 | 0.582304 | 0,08 (-0,21 ; 0,36) |
| JAK2 V617F% | RBC T/L | 43 | 103 | 0.061581 | 0,18 (-0,01 ; 0,37) |
| JAK2 V617F% | HB mmol/L | 18 | 128 | 0.601824 | -0,05 (-0,22 ; 0,13) |
| JAK2 V617F% | HT L/L | 26 | 120 | 0.198133 | 0,12 (-0,07 ; 0,3) |
| JAK2 V617F% | MCV fL | 43 | 103 | 0.080998 | -0,17 (-0,36 ; 0,03) |
| JAK2 V617F% | MCH HB/RBC fmol | 43 | 103 | *0.003781* | ***-0,28 (-0,46 ; -0,09)*** |
| JAK2 V617F% | MCHC HB/HT mmol/l | 29 | 117 | *0.023141* | ***-0,21 (-0,38 ; -0,02)*** |
| JAK2 V617F% | RDW % | 44 | 102 | *0.000024* | ***0,41 (0,22 ; 0,56)*** |
| JAK2 V617F% | PLT G/L | 18 | 128 | *0.030169* | ***-0,19 (-0,36 ; -0,01)*** |
| JAK2 V617F% | MPV fL | 49 | 97 | 0.689597 | 0,04 (-0,17 ; 0,24) |

Supplementary Figure 1
Myelofibrosis free survival time in PV patients studied (n=71)
a) whole group b) pts with co-existing non-driver variants, and according to c) *JAK2* rs12343867 d) *TERT* rs2736100 e) *OBFC1* rs9420907 and f) *miR-146a* rs2431697 genotypes status

# a) whole PV group b) additional variants (0-no, 1-yes) p=0.3694 c) *JAK2* rs12343867 p=0.5609

# *
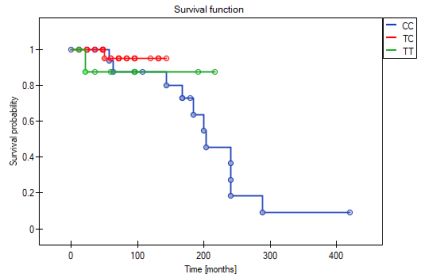
*
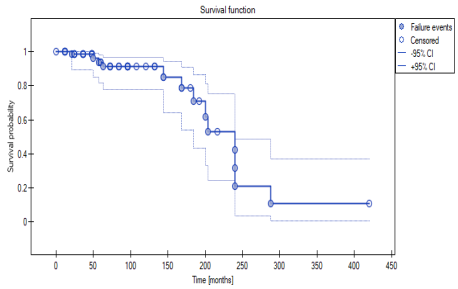


# *
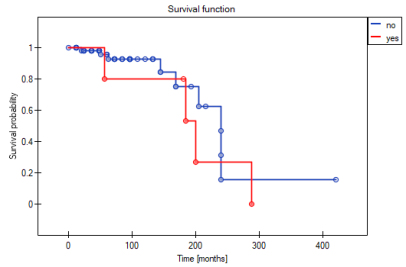
*

# d) *TERT* rs2736100 p=0.4759 e) *OBFC1* rs9420907 p=0.5485 f) *miR-146a* p=0.3024

# *
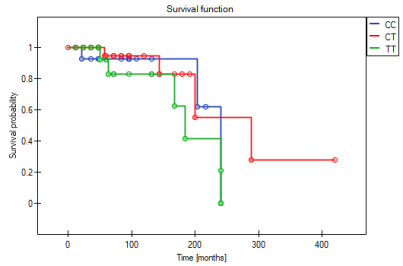

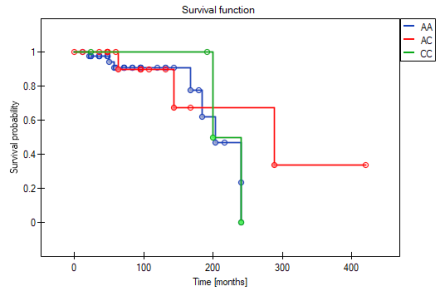

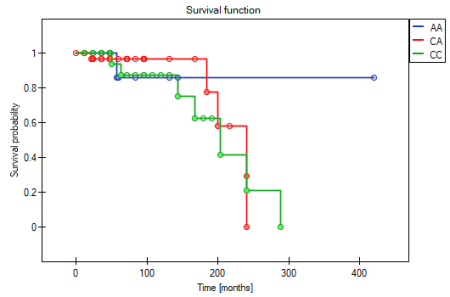
*

Supplementary Figure 2
The impact of studied SNV’s and presence of additional non-driver variants on overall survival of patients with polycythaemia vera.
OS in a) whole group (data available n=142) b) patients without and with additional variants and according to c) *JAK2* rs12343867 d) *TERT* rs2736100 e) *OBFC1* rs9420907 and f) *miR-146a* rs2431697 status

# a) whole PV group b) additional variants (0-no, 1-yes) p=0.9355 c) *JAK2* rs12343867 p=0.3425


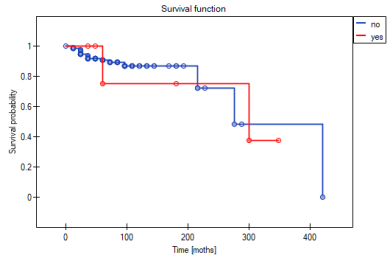
**
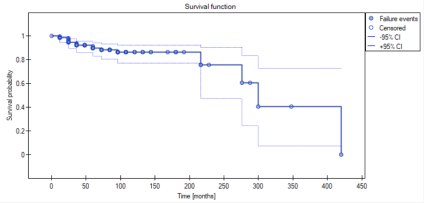
**

**
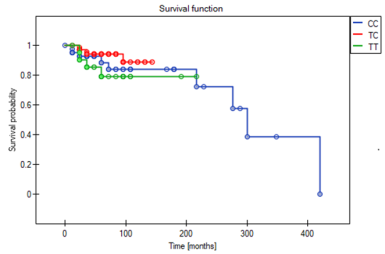
**

# d) *TERT* rs2736100 p=0.8881 e) *OBFC1* rs9420907 p=0.6966 f) miR-146a p=0.5355


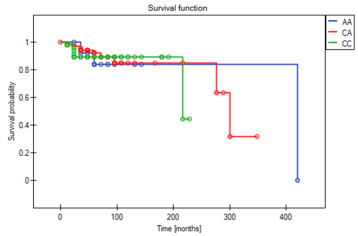

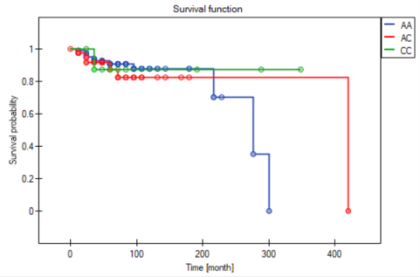

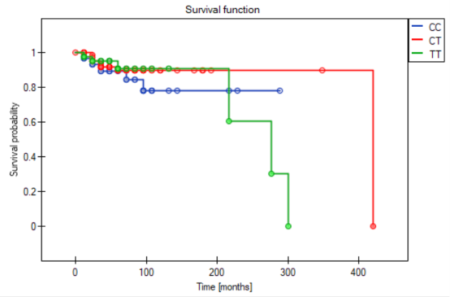

Supplement: Supplementary file 1 — Supplementary file1 (DOCX 307 KB) [file 432_2022_4327_MOESM1_ESM.docx]
